# HTLV-1 affects the adipogenic differentiation of human mesenchymal stem cells

**DOI:** 10.1186/1742-4690-8-S1-A115

**Published:** 2011-06-06

**Authors:** Evandra Strazza Rodrigues, Maurício C Rocha, Maristela Delgado Orellana, Osvaldo Massaiti Takayanagui, Dimas Tadeu Covas, Simone Kashima

**Affiliations:** 1 Regional Blood Center of Ribeirão Preto, Ribeirão Preto, Brazil; 2 Faculty of Pharmaceutical Sciences of Ribeirão Preto, University of São Paulo, Ribeirão Preto, Brazil; 3 Faculty of Medicine of Ribeirão Preto, University of São Paulo, Ribeirão Preto, Brazil

## Background

Mesenchymal stem cells (MSCs) are multipotent cells mainly found in the bone marrow (BM) and are considered to support human hematopoiesis. During HTLV-1 infection, T cells that are infected may migrate to the BM, and establish a progressive viral invasion. The persistence of these cells in the BM may affect the function of the resident BM cells and the biological properties of this microenvironment. However, the exact mechanisms of this phenomenon and the relative contribution of HTLV-1 to BM cell dysfunction remain to be elucidated. In this study, we evaluated the ability of HTLV-1 to disturb MSCs normal functions.

## Methods

Human MSCs were cultured on the presence of MT-2 cell line. MSCs markers and adipogenic differentiation potential was evaluated.

## Results

We observed that MSCs that had contact with HTLV-1 were able to differentiate into adipocytes. However, gene expression of PPARG, cell morphology and intracellular lipid formation analyses revealed that adipogenic differentiation of MSCs that were exposed to HTLV-1 was decreased compared to MSCs not exposed (p<0.03). In addition, analyses by flow cytometry showed a reduction of the expression of MSCs markers (CD271 and HLA-DR) in MSCs that had contact with HTLV-1.

## Conclusion

HTLV-1 caused significant changes in the characteristics and functions of MSCs. This finding suggests the importance of HTLV infection on MSCs, and this could contribute to the understanding the viral infection mechanisms and pathogenesis.

